# Coordinated regulation of acid resistance in *Escherichia coli*

**DOI:** 10.1186/s12918-016-0376-y

**Published:** 2017-01-06

**Authors:** Patricia Aquino, Brent Honda, Suma Jaini, Anna Lyubetskaya, Krutika Hosur, Joanna G. Chiu, Iriny Ekladious, Dongjian Hu, Lin Jin, Marianna K. Sayeg, Arion I. Stettner, Julia Wang, Brandon G. Wong, Winnie S. Wong, Stephen L. Alexander, Cong Ba, Seth I. Bensussen, David B. Bernstein, Dana Braff, Susie Cha, Daniel I. Cheng, Jang Hwan Cho, Kenny Chou, James Chuang, Daniel E. Gastler, Daniel J. Grasso, John S. Greifenberger, Chen Guo, Anna K. Hawes, Divya V. Israni, Saloni R. Jain, Jessica Kim, Junyu Lei, Hao Li, David Li, Qian Li, Christopher P. Mancuso, Ning Mao, Salwa F. Masud, Cari L. Meisel, Jing Mi, Christine S. Nykyforchyn, Minhee Park, Hannah M. Peterson, Alfred K. Ramirez, Daniel S. Reynolds, Nae Gyune Rim, Jared C. Saffie, Hang Su, Wendell R. Su, Yaqing Su, Meng Sun, Meghan M. Thommes, Tao Tu, Nitinun Varongchayakul, Tyler E. Wagner, Benjamin H. Weinberg, Rouhui Yang, Anastasia Yaroslavsky, Christine Yoon, Yanyu Zhao, Alicia J. Zollinger, Anne M. Stringer, John W. Foster, Joseph Wade, Sahadaven Raman, Natasha Broude, Wilson W. Wong, James E. Galagan

**Affiliations:** 1 Department of Biomedical Engineering, Boston University, Boston, USA; 2 Bioinformatics program, Boston University, Boston, USA; 3 BE605 Course, Biomedical Engineering, Boston University, Boston, USA; 4 Wadsworth Center, New York State Department of Health, Albany, NY USA; 5 Department of Biomedical Sciences, University at Albany, Albany, NY USA; 6 Department of Microbiology and Immunology, University of South Alabama College of Medicine, Mobile, AL 36688 USA; 7 National Emerging Infectious Diseases Laboratory, Boston University, Boston, USA

**Keywords:** Acid resistance, Regulatory network modeling, Systems biology/ChIP-Seq

## Abstract

**Background:**

Enteric *Escherichia coli* survives the highly acidic environment of the stomach through multiple acid resistance (AR) mechanisms. The most effective system, AR2, decarboxylates externally-derived glutamate to remove cytoplasmic protons and excrete GABA. The first described system, AR1, does not require an external amino acid. Its mechanism has not been determined. The regulation of the multiple AR systems and their coordination with broader cellular metabolism has not been fully explored.

**Results:**

We utilized a combination of ChIP-Seq and gene expression analysis to experimentally map the regulatory interactions of four TFs: *nac*, *ntrC*, *ompR*, and *csiR*. Our data identified all previously *in vivo* confirmed direct interactions and revealed several others previously inferred from gene expression data. Our data demonstrate that *nac* and *csiR* directly modulate AR, and leads to a regulatory network model in which all four TFs participate in coordinating acid resistance, glutamate metabolism, and nitrogen metabolism. This model predicts a novel mechanism for AR1 by which the decarboxylation enzymes of AR2 are used with internally derived glutamate. This hypothesis makes several testable predictions that we confirmed experimentally.

**Conclusions:**

Our data suggest that the regulatory network underlying AR is complex and deeply interconnected with the regulation of GABA and glutamate metabolism, nitrogen metabolism. These connections underlie and experimentally validated model of AR1 in which the decarboxylation enzymes of AR2 are used with internally derived glutamate.

**Electronic supplementary material:**

The online version of this article (doi:10.1186/s12918-016-0376-y) contains supplementary material, which is available to authorized users.

## Background

*Escherichia coli* can act as both a commensal and potential pathogen. Pathogenic strains of *E. coli* cause a range of diseases including urinary tract infections, pneumonia, meningitis, and enteric infections. Survival of enteric *E. coli* strains requires contending with the highly acidic environment of the human digestive tract. The stomach, with a pH as low as 1.5, provides protection against microbial infection. *E. coli* is known to be unusually tolerant to acid, rivaling the tolerance of *Helicobacter pylori* [1–3]. This tolerance may contribute to the unusually low dose required for an *E. coli* infection, requiring only ~10^2^ cell dosage for infection [4–10]. Thus, although not a virulence factor specific to pathogenesis, innate acid adaptation systems are nonetheless essential for the survival of both pathogenic and non-pathogenic enteric *E. coli* [3, 11–13].

Multiple acid resistance (AR) mechanisms have been described for *E. coli* [1–3, 7, 11, 14]. Four of the five primary systems utilize a pyridoxal-5’-phosphate (PLP)-dependent amino acid decarboxylase with an externally derived amino acid to consume a proton and generate a by-product and CO_2_. A corresponding anti-porter exchanges the amino acid and by-product across the membrane. The glutamate-dependent system named as AR2 or GDAR, is the most robust system, allowing up to 80% survival after 2 h in extremely low pH and producing GABA as by-product [11]. Other amino acid dependent systems are the arginine-dependent system (AR3 or ADAR) the lysine-dependent system (AR4 or LDAR), and the more recently discovered ornithine-dependent system (ODAR) [15]. The first described AR system, AR1, is an oxidative AR system repressed by glucose that is σ^S^-dependent and does not require an externally-derived amino acid [2, 4, 11]. Despite the fact that AR1 was the first discovered AR system, its mechanism has still not been determined.

The main transcriptional regulatory elements of amino acid-dependent AR have been characterized. GadE is the primary regulator of AR2 and serves as a transcriptional activator for genes encoding two glutamate decarboxylase isoforms (*gadA* and *gadB*) and the glutamate/GABA antiporter (*gadC*) [16–18]. Transcriptional activation of *gadA*/*B* requires heterodimerization of GadE with RcsB [19]. Regulation of *gadE*, in turn, is complex and involves the activities of multiple circuits whose effects are integrated by binding to the large intergenic region upstream of *gadE* [2, 16, 20, 21]. AdiY is the primary regulator of AR3 that, together with CysB, coordinately regulates the corresponding arginine decarboxylase gene (*adiA*) [22]. CadC is the primary regulator of AR4, regulating both the lysine decarboxylase (*cadA*) and antiporter (*cadB*) genes [23]. The regulatory network for ODAR is not well-defined. With the exception of the binding of GadE-RcsB to the *cadBA* promoter, it is not known whether or how the regulation different system AR systems and adaptations are coordinated. Elements of AR2 can be induced by non-acid stimuli including treatment with acetate and entry into stationary phase [11]. In addition, acid stress leads to adaptations beyond the amino acid-dependent AR machinery including expression of the electron transport chain, the envelope stress response and alterations in membrane permeability to protons, a formate hydrogen lyase system that reduces protons to hydrogen gas, and reversals in the cell potential that may drive a chloride/proton antiporter, and numerous metabolic processes [1, 2, 24–26]. The regulatory mechanisms underlying these expression changes have not been established, and the coordination of these and other acid responses with broader cellular metabolism has not been fully explored.

Despite extensive mapping of genes and their regulatory elements in *E. coli*, only a small fraction of its TFs have been studied on a genomic scale. Chromatin-immunoprecipitation followed by sequencing (ChIP-Seq) enables genome-wide mapping of TF binding sites and has been applied extensively to eukaryotes [27]. In every organism in which ChIP-Seq has been applied, many more binding sites for even well studied TFs have been reproducibly discovered [28]. Surprisingly, these approaches have been used sparingly in *E. coli*. ChIP-microarray (or ChIP-chip) data has been described for only 19 of 297 *E. coli* TFs while higher resolution ChIP-Seq and ChIP-exo data have been described for only a handful [29–39]. In bacteria, ChIP-Seq identifies binding sites with high reproducibility and spatial resolution frequently sufficient to identify multiple binding sites within a single promoter but cannot establish if these sites have functions [40–42]. The integration of ChIP-Seq and gene expression data following TF perturbations allows us to identify binding sites that have putative regulatory effects, distinguishing between direct and indirect regulatory effects. This approach has been used to map the transcriptional regulatory network for *Mycobacterium tuberculosis* (MTB) [42] and networks in *S*. Typhimurium [43, 44] and *E. coli* [31].

As part of an on-going effort to comprehensively map the transcriptional regulatory network of *E. coli*, we have performed ChIP-Seq on a large number of *E. coli* TFs. We report here the results for 4 TFs with interactions relevant to AR: CsiR, Nac, NtrC, and OmpR. We identified all previously reported *in vivo* direct interactions for these TFs and confirmed several others previously inferred from gene expression data. Our data further demonstrated that *nac* and *csiR* directly modulate AR, and lead to a regulatory network model in which all four TFs participate in coordinating acid resistance, glutamate metabolism, and nitrogen metabolism. This model predicts a novel mechanism for AR1 by which the decarboxylation enzymes of AR2 are used with internally derived glutamate. This hypothesis makes several testable predictions that we confirmed experimentally.

## Methods

### Bacterial strains and culture conditions

Single gene knock-out strains (*Δnac*, *ΔcsiR*, *ΔgadC*, *ΔgadE*, *ΔgadA*, *ΔgadB*) were obtained from the Keio collection and verified via PCR. The *ΔgadAΔgadB KO* and the *ΔgabDTP*/*ΔcsiR KO* was created by using the one step gene-inactivation technique by Datsenko and Wanner [45] using *ΔgadA* and *ΔcsiR* as a background strains respectively. Cells are grown and maintained in LB media with kanamycin and chloramphenicol. For wild-type, *E. coli* strain K-12 MG1655 was used.

### ChIP-Seq

TFs were ligated into pT7-FLAG-4 vector (Sigma-Aldrich) for Flag-tagging and inducible expression. Plasmids were cloned into *E. coli* MG1655 strains and checked for kanamycin selection. Fidelity of the clones were validated through sequencing. Western blot verified production of inducible Flag-tagged TF using 1 mM IPTG. ChIP assays were performed by induction of strains in LB media starting at OD_600_ 0.2 with 1 mM IPTG for 2 h. Cells were fixed with formaldehyde and glycine and sheared through sonication before immunoprecipitation with anti-FLAG monoclonal antibody. Further pull-down was done using agarose protein G beads. Reverse cross-linking of samples was performed by incubation with Proteinase K. DNA purification was carried out using DNA purification kit (Qiagen). Library preparation was done using standard Illumina TruSeq ChIP Sample Preparation protocols. ChIP replicate experiments presented here were performed by students as part of final projects for course BE605 in Biomedical Engineering at Boston University. Multiplexed sequencing was performed on an Illumina GAIIx Sequencer that generated single 50 bp reads. Total reads generated for the sequencing runs ranged from 3.5 – 22 million reads with an average of 10.62 million reads. ChIP-Seq control samples were wild-type strains with and without empty vectors subjected to the same immunoprecipitation protocol.

### ChIP-qPCR

40 ml *E. coli* cells expressing C-terminally FLAG-tagged Nac were grown in Gutnick Medium [46] at 30 °C and supplemented with 2 mM NH_4_Cl. Cultures were harvested 60 min after growth ceased (nitrogen depleted), at an OD_600_ between 0.6 and 0.7. ChIP was performed as previously described [35]. To serve as an “input” control, 20 μl chromatin were also de-crosslinked by boiling for 10 min and cleaned up using a PCR purification kit (Qiagen). ChIP and input samples were analyzed using an ABI 7500 Fast real time PCR machine. Enrichment of ChIP samples was calculated relative to a control region within the transcriptionally silent *bglB* gene and normalized to input DNA. Occupancy units represent background-subtracted fold-enrichment.

### RNA-Seq

RNA-Seq was performed following induction of Nac and CsiR using the same TF inducible *E. coli* strains used in ChIP-Seq as described above. Control experiments under identical conditions were also performed on WT *E. coli*. 50 mL of TF-inducible strains were induced with 1 mM IPTG for 2 h starting at OD_600_ 0.2 in LB media. Total RNA extraction was performed using TRIzol® reagent (LifeTechnologies). Samples were subjected to 1-h DNAse digestion and purified using RNeasy spin columns (Qiagen). Samples were processed using Ribo-Zero rRNA removal kits and library preparation was done using NEB Next ultra-directional RNA library prep kit for Illumina. Multiplexed sequencing was performed on an Illumina GAIIx Sequencer that generated single 40 bp reads. Total coverage for the sequencing runs ranged from 8–14 million reads with an average of 10 million reads.

### OmpR RT-PCR

50 mL of the *ompR*-inducible strains were grown in LB media starting at OD_600_ 0.2 with 1 mM IPTG for 2 h. For *ΔcsiR* and *Δnac* strains, strains were subject to AR2 acid challenge conditions described below. Total RNA extraction was performed using TRIzol® reagent (LifeTechnologies). Samples were subjected to 1-h DNAse digestion and purified using RNeasy spin columns (Qiagen). Samples were analyzed using BioRad CFX96 Real-Time System C1000 Thermal Cycler. Gene expression was calculated using the ΔΔC_t_ method with rpoD as a reference gene.

### Data analysis

The analysis of ChIP-Seq data to identify binding sites was performed as previously described [28, 42, 47]. Reads were aligned to *E. coli* genome (Genbank entry U00096.2). Binding sites were compared to reported binding sites from EcoCyc [48] after manually curating reported regulatory interactions for those with experimental evidence for binding (Additional file 1: Table S1). Binding sites were assigned to potential gene targets based on proximity to potential promoters and taking into account operon structure from EcoCyc. Genes with start codons within 500 bp of a binding site were considered as potential targets. In the case of divergent promoters the gene closest to the binding site was considered to be the target unless gene expression data or known promoter structure indicated an alternative target or potential regulation of both divergently transcribed genes.

Determination of binding sequence motifs was performed using MEME SUITE tool (version 4.10.2) [49]. A 4th-order markov model based on the whole genome sequence served as background bfile to create more accurate motifs.

For the analysis of RNA-Seq data, Bowtie2 [50] was used to align raw reads to the *E. coli* genome (Genbank entry U00096.2) and samtools [51] was used to obtain BAM files. R scripts (Bioconductor GenomicRanges [52] package and custom-written scripts) were used to calculate raw read counts per gene and RPKMs. Differential expression was calculated as the ratio of RPKMs after TF induction to RPMKs in control experiments with WT *E. coli*.

### Acid challenge assays

Acid challenge (AR) assays were all adapted from the protocol described by Castanie-Cornet et al. [11].

### Testing AR1

Cultures are grown overnight in LB media buffered at pH 5.5 with 100 mM mopholinethanesulfonic acid (MES) at 37 °C. A negative control sample was also cultured overnight in EG media at pH 7.0 in 37 °C. New 1:1000 diluted test cultures are made in E-minimal media with 0.5% glucose (EG media) adjusted to pH 2.5 and pH 7.0 respectively while the negative control was diluted by 1:1000 into EG-media at pH 2.5. All diluted cultures were incubated for 2 h in 37 °C and were then plated in LB plates. The number of colony-forming units (CFUs) after overnight plate incubation at 37 °C were counted to determine survival. Kanamycin (50 μg/mL) was added to media for the knockout strains.

### Testing AR2/GDAR

Cultures are grown overnight in LB media with 0.5% glucose at pH 7.0 in 37 °C. New 1:1000 diluted test cultures are made in E-minimal media with 0.5% glucose (EG media) at pH 2.5 supplemented with 1 mM L-glutamate. A negative control culture was also prepared without L-glutamate supplement. Test cultures were incubated for 2 h in 37 °C and were then plated in LB plates. The number of colony-forming units (CFUs) after overnight plate incubation at 37 °C were counted to determine survival. Kanamycin (50 μg/mL) was added to media for the knockout strains.

### Induction of gadE for AR rescue

GadE was cloned into a pZE11 expression vector under the control of the pLtetO promoter [53]. This construct was transformed into WT, Δ*csiR*, Δ*nac* and Δ*gadE* strains. GadE induction was carried out by addition of anhydrotetracycline (aTc) during the incubation at 37 °C step. Acid challenge was performed according to procedure above.

### For nac and ntrC physiological induction

*E. coli* MG1655 WT strains were grown on N^−^C^−^ minimal media supplemented with 0.4% glucose to mid-exponential phase (OD_600_ = 0.5). 5 mM glutamine was added as control [54].

### For csiR natural physiological induction

*E. coli* MG1655 WT strains were grown on LB media to lag and mid-exponential phases (OD_600_ 0.1 and 0.5 respectively). The sample in lag phase served as control [55].

### For ompR physiological induction

*E. coli* MG1655 WT strains were grown on LB media mid-exponential phase (OD_600_ = 0.5) with 20% sucrose. A sample without sucrose was used as control [56].

### For TF artificial induction

Inducible TF strains were incubated in LB media at 37 °C with 1 mM IPTG for 2 h.

### RNA extraction and qRT-PCR

For the samples in the above section, total RNA extraction was performed using RNeasy Protect Bacteria kit (Qiagen). Samples were subjected to 1-h TURBO DNAse digestion and purified using AMPure RNAclean XP beads. qRT-PCR was performed using BioRad CFX96 Real-Time System C1000 Thermal Cycler using gene-specific primers. Gene expression was calculated using the ΔΔC_t_ method with rpoD serving as a reference gene.

## Results

### Validation of Binding Site Mapping

Our regulatory network mapping strategy utilized transcription factors tagged with FLAG and under inducible control (Methods) [28, 42, 57–60]. Importantly, control ChIP-seq experiments in strains lacking FLAG-tagged proteins revealed minimal non-specific binding in *E. coli*. The use of an inducible promoter system ensures expression of targeted TFs, which allowed us to study the binding of all TFs in the same standard reproducible condition. While the induction of TFs raises potential concerns about overexpression artifacts, we confirmed the accuracy of this approach in *E. coli* for the TFs studied in this report in several ways. First, we identified all previously experimentally validated *in vivo* direct interactions from EcoCyc [48] with high spatial accuracy (Additional file 1: Table S1 and Table S2). Second, motifs inferred from our binding data are consistent with those previously described (Additional file 1: Figure S1). Third, our data for NtrC are consistent with previously published data for NtrC induced from its native promoter [30] (Additional file 1: Figure S3). Finally, our results for binding site accuracy in *E. coli* are consistent with the results of TF mapping in *Mycobacterium tuberculosis* and related *Mycobacteria* [42, 57–59].

### Analysis of Regulatory Interactions

Our ChIP-Seq data identify a large number of previously undetected binding sites (Additional file 1: Table S1, Additional files 2, 3, 4 and 5) including binding over a range of coverage enrichment, potentially reflecting differences in binding affinity [42]. In addition, although binding within intergenic regions is enriched over what would be expected by chance, a large number of binding sites in genes were also identified. This has been commonly reported for other ChIP-Seq studies in bacteria [28, 47]. To assess the potential transcriptional functions of these newly identified binding sites, we analyzed transcriptomic data following the perturbation of each TF (Additional files 2 and 3). For Nac and CsiR, we performed RNA-Seq after TF induction using the same strains used for ChIP-Seq and present the genes most likely affected based on our binding and expression data (see Tables 1 and 2, Methods). We also analyzed previously published microarray data for an *E. coli* strain in which a mutation in the NtrC-activating kinase, NtrB, upregulates NtrC [54]. This publication compared microarray data for NtrC upregulation to an *ntrC* deletion strain. Since induction of NtrC also induces *nac*, these data reveal genes directly or indirectly induced by both TFs. We did not assess the impact of OmpR on RNA levels genome-wide, but rather we performed gene-specific RT-PCR. We also performed RT-PCR following TF perturbations to validate additional specific interactions, as described below. Using the combination of ChIP-Seq and transcriptomic data, we identified potential direct regulatory interactions as described in the Methods. We first describe our results in detail for each TF, and then describe a global regulatory network arising from this analysis that links acid resistance with central metabolism.

**Table 1:** Summarized list of most affected genes from induced TF RNA-Seq data with corresponding ChIP-Seq binding sites

Gene symbol	EcoCyc locus	ChIP-Seq peak location	Type	Fold-change (FC)	Log _2_ (FC)
Nac-induced RNA-Seq					
*Top 20 over*-*expressed genes*					
nac	EG14265	2059466	genic	187.159	7.548
yfgG	EG14203	2627183	intergenic	138.340	7.112
pyrL	EG11279	4470803	intergenic	98.660	6.624
ileY	EG31121	2783527	intergenic	87.740	6.455
shoB	EG14494	2697790	genic	82.380	6.364
ilvL	EG11270	3948282	intergenic	67.590	6.079
nrfF	EG11949	4291501	genic	52.500	5.714
ybgE	EG12395	773855	intergenic	45.840	5.519
yghG	EG12991	3111175	genic	33.080	5.048
ynaK	EG14296	1423084	genic	25.970	4.699
allR	EG13616	532179	intergenic	17.160	4.101
nanK	EG12815	3368556	genic	16.050	4.005
wcaE	EG13573	2128058	genic	9.820	3.296
rfbC	EG11979	2108210	genic	8.854	3.146
yqeJ	EG13101	2987333	genic	7.346	2.877
cmtA	EG11792	3076545	genic	5.750	2.524
hcaE	EG13456	2666608	genic	5.032	2.331
yqiC	EG13031	3183243	intergenic	4.741	2.245
yqeH	EG13099	2985944	genic	4.544	2.184
eutS	EG14192	2574048	intergenic	3.761	1.911
*Top 20 repressed genes*					
yhfL	EG12907	3497156	genic	0.008	−6.928
chpS	EG11250	4446394	intergenic	0.012	−6.349
leuU	EG30050	3320495	genic	0.013	−6.299
fepE	EG10297	617863	genic	0.013	−6.295
bfd	EG11181	3464917	genic	0.014	−6.137
yhiJ	EG12225	3631010	genic	0.017	−5.891
yfhL	EG13215	2697790	genic	0.019	−5.723
scpB	EG12972	3062091	genic	0.019	−5.718
ampD	EG10041	118719	intergenic	0.020	−5.644
yafN	EG13151	252250	genic	0.021	−5.555
ybbC	EG11769	526792	intergenic	0.027	−5.235
yafO	EG13152	252250	genic	0.029	−5.125
ccmA	EG12059	2295447	genic	0.030	−5.067
yggP	EG12976	3075011	genic	0.036	−4.779
iraP	EG11256	400152	intergenic	0.037	−4.750
pabC	EG11493	1152528	genic	0.038	−4.703
rfbB	EG12412	2110788	genic	0.039	−4.695
macA	EG13694	918441	intergenic	0.040	−4.658
hfq	EG10438	4398299	intergenic	0.044	−4.517
yegR	EG14061	2165875	intergenic	0.045	−4.477
CsiR-induced RNA-Seq					
*Top 20 over*-*expressed genes*					
yehD	EG11990	2190601	genic	55.750	55.750
yjjP	EG12592	4601377	intergenic	23.030	23.030
ygiW	EG13025	3167234	genic	5.438	5.438
dinI	EG12670	1120353	intergenic	4.335	4.335
ychQ	EG14293	1265792	genic	4.208676729	4.208676729
bssS	EG14335	1120353	intergenic	4.146456347	4.146456347
gadX	EG12243	3663762	genic	3.958	3.958
yfbU	EG14105	2410409	genic	3.063536927	3.063536927
ppiB	EG10758	553885	genic	2.985	2.985
orn	EG12480	4,389,621	intergenic	2.979	2.979
gltF	EG11514	3358941	intergenic	2.470	2.470
murD	EG10620	97136	genic	2.419	2.419
pliG	EG13892	1,226,238	intergenic	2.328431905	2.328431905
hinT	EG12172	1160988	intergenic	2.280	2.280
ebgC	EG10253	3223817	genic	2.159	2.159
yfcV	EG14125	2454000	intergenic	1.865631986	1.865631986
rfbB	EG12412	2110925	genic	1.850	1.850
ydeO	EG13797	1581558	genic	1.836799927	1.836799927
smg	EG11605	3430204	genic	1.748	1.748
yfdV	EG14144	2488614	genic	1.644414859	1.644414859
*Top 20 repressed genes*					
yobD	EG13948	1903280	genic	0.164030959	−2.607959959
yihM	EG11839	4059288	genic	0.186	−2.423
csiD	EG13523	2786890	intergenic	0.245517248	−2.026103713
ycjP	EG13913	1372194	genic	0.269455258	−1.891882356
yccU	EG13723	1027171	genic	0.275398754	−1.860406061
yjdP	EG14407	4311501	genic	0.282972057	−1.8212685
fhuE	EG10306	1160988	intergenic	0.326	−1.617
mrdA	EG10606	667202	genic	0.371	−1.429
yfcO	EG14118	2447860	genic	0.381433124	−1.390497962
baeS	EG11617	2160863	genic	0.389	−1.360
yfbP	EG14100	2386855	genic	0.39350482	−1.345546788
bdcR	EG12529	4471822	genic	0.404	−1.306
ycbU	EG13713	1002250	genic	0.433672161	−1.205323262
ydfI	EG13821	1629426	genic	0.439353983	−1.186544321
ttdR	EG12694	3204662	genic	0.440	−1.185
yfbT	EG14104	2410409	genic	0.444846984	−1.168618925
dinQ	EG14431	3645540	genic	0.458480805	−1.12506676
yciU	EG14256	1304868	genic	0.463635491	−1.108937087
gcvA	EG11795	2940361	genic	0.484	−1.046
oxc	EG14143	2490338	genic	0.486938022	−1.038189938

**Table 2:** Selected list of combined ChIP-Seq and RNA-Seq data for AR-related genes following induction of nac and csiR showing direct regulatory effect

Gene symbol	EcoCyc locus	ChIP-Seq peak location	Type	Fold-change (FC)	Log _2_ (FC)
Nac-induced RNA-Seq					
gadE	EG11544	3656717	genic	3.321	1.732
sdhC	EG10933	753984	intergenic	2.494	1.318
evgA	EG11609	2481403	intergenic	0.288	−1.797
ompR	EG10672	3534783	intergenic	0.894	−0.161
CsiR-induced RNA-Seq					
gadX	EG12243	3663762	genic	3.958	1.985
ydeO	EG13797	1581558	genic	1.837	0.877
gadW	EG12242	3662685	intergenic	0.728	−0.458
gdhA	EG10372	1840440	genic	0.522	−0.937

#### CsiR

**Fig. 1:**
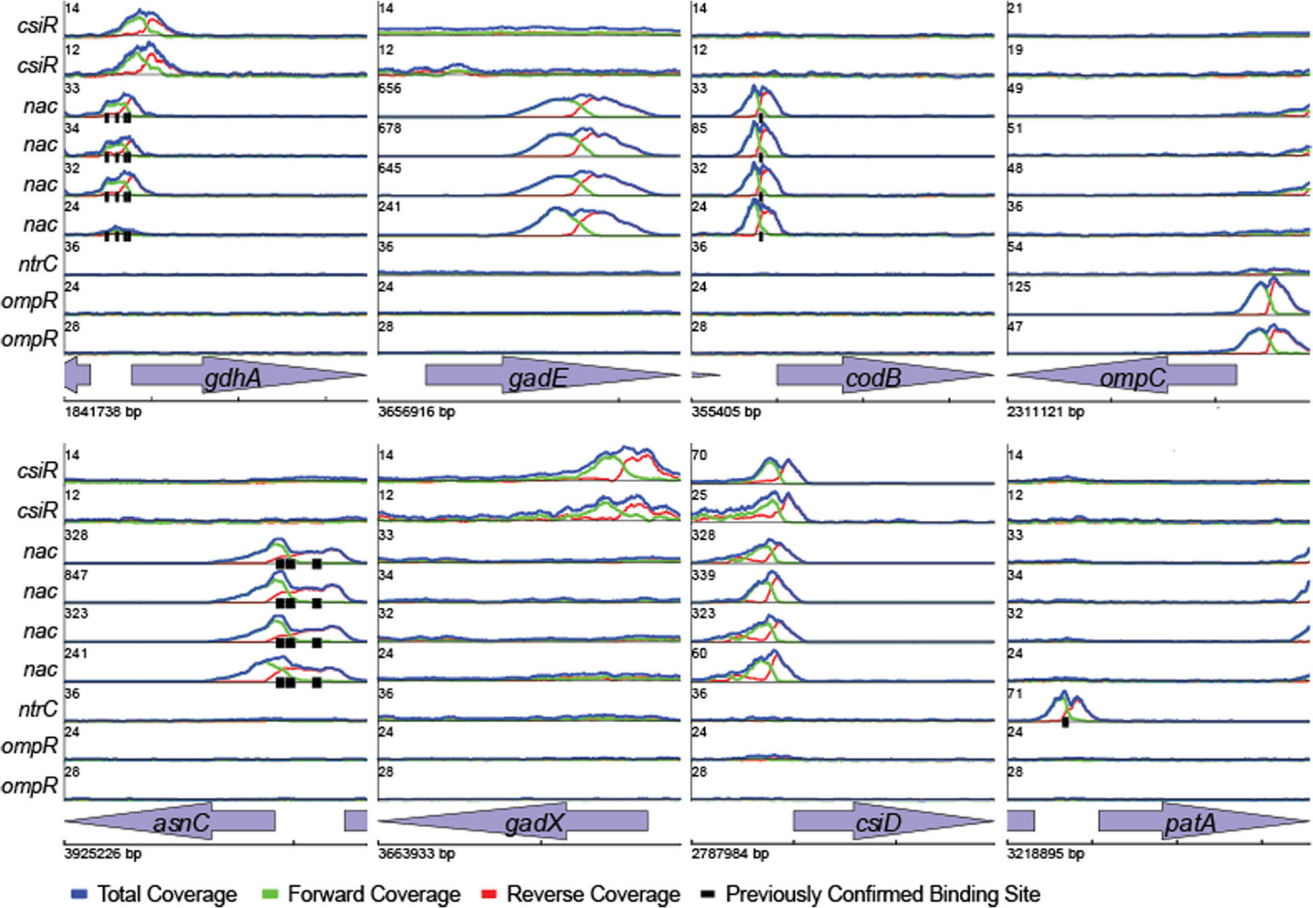
Mapping *E. coli* transcriptional regulatory interactions using ChIP-Seq. Examples of identified binding sites for csiR, nac, ntrC, and ompR. Each panel plots the total read coverage (*blue*), forward read coverage (*green*), and reverse read coverage (*red*). The maximum coverage for each plot is given by the number on the y-axis in units of coverage normalized to mean coverage. Multiple biological replicate experiments are shown for 3 TFs as noted on leftmost y-axes. ChIP-Seq coverage plots are shown for 8 separate genomic regions. The start location of each region is provided at the bottom left x-axes. The tick marks on the bottom x-axes are spaced 500 bp apart. Different regions are plotted at different scales for clarity. Previously described binding sites from EcoCyc are shown as black ticks below the coverage plot in each panel

**Fig. 2:**
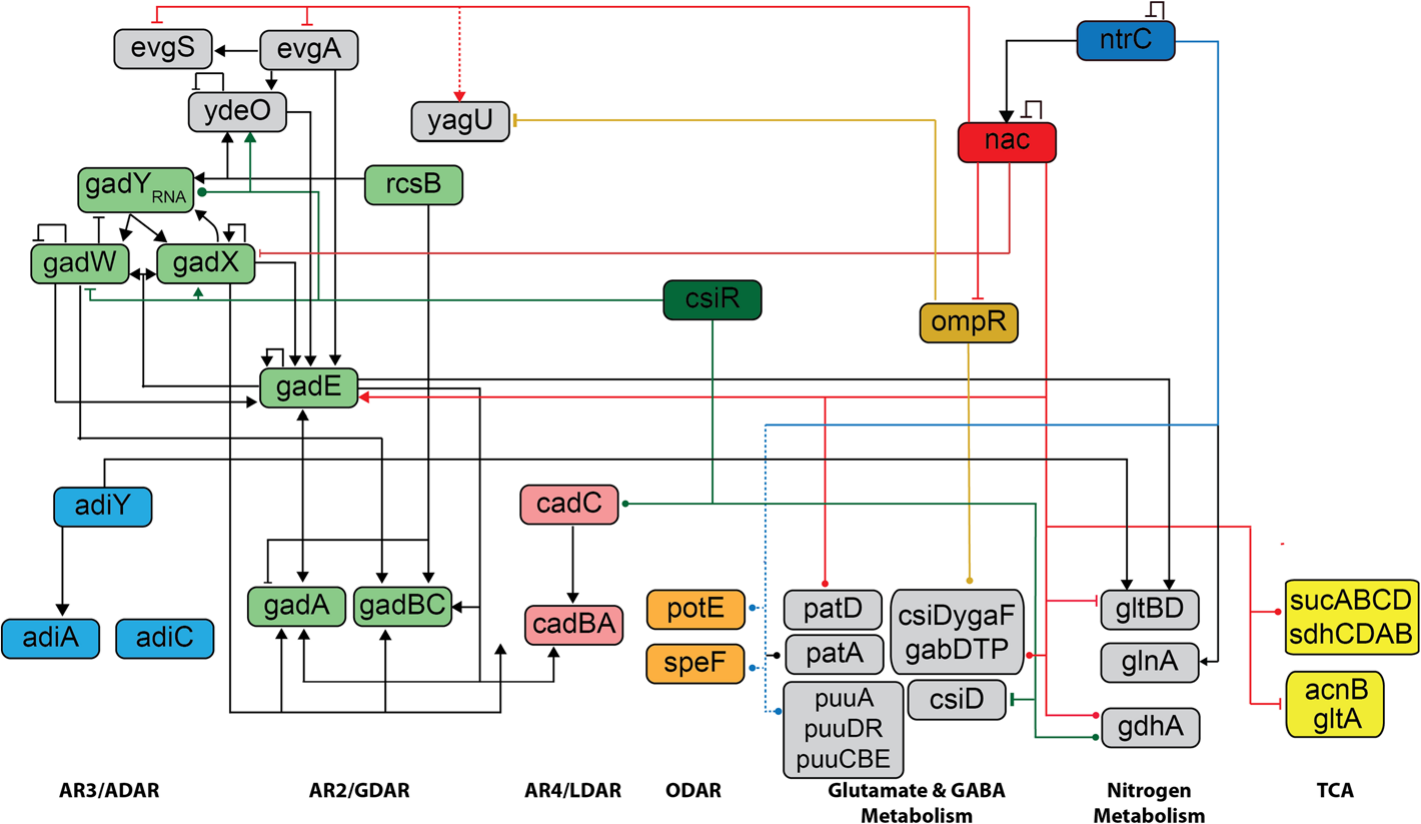
ChIP-Seq mapping and transcriptomics reveal regulatory links between AR systems and cellular metabolic pathways. Map of selected direct binding sites potentially associated with AR. Novel TF binding is displayed as colored dashed lines. Novel regulatory links confirmed with gene expression data are shown as solid colored lines. Black lines signify previously reported known binding and regulation. Circle terminators indicate unconfirmed or indeterminate regulatory effect

CsiR is reported to repress *csiD* via a σ^s^ promoter upstream of the *csiD*-*ygaF*-*gabD*-*gabT*-*gabP* operon, though mutation of *csiR* does not directly impact regulation of *gabDTP*, potentially due to two internal promoters near *gabD* [55]. GabDTP are involved in the inter-conversion of GABA and alpha-ketoglutarate (α-KG). It has been postulated that these genes may play a role in linking acid resistance to the TCA cycle (Additional file 1: Figure S5) through the metabolism of GABA derived from AR2, although this has not been confirmed [55]. Moreover, no direct binding of CsiR to any operon has been reported [55, 61]. Our ChIP-Seq data confirms the expected binding site for CsiR in the *csiD* promoter (Fig. 1). Consistent with previous results, CsiR induction represses *csiD* while no significant expression changes were observed for *ygaF* or *gabDTP*. Our data also reveal a surprising number of novel binding sites of potential relevance to AR (see Table 2). We observe binding to *gadX* and *ydeO*, and RNA-Seq following CsiR induction indicates strong activation of both. CsiR also binds to the divergent promoter between *gadW* and *gadY*. We also observe weak repression of *gadW* and no evident effect on *gadY*, suggesting that this binding site operates on the *gadW* promoter (Additional file 2 ). GadX, YdeO, GadW and GadY are four regulators that form a complex circuit capable of activating the core AR2 genes *gadE*, *gadA*, *gadB*, and *gadC* (Fig. 2) [62, 63]. We see strong activation of all of these AR2 genes after induction of CsiR (see Table 2). We also observe that CsiR binds to the AR4 regulator *cadC*. No significant effect of *csiR* induction on *cadC* was evident in our data, though *cadBA* was moderately repressed. We further observe that induction of CsiR results in repression of the AR3 genes *adiY* and *adiA*, though this appears to reflect indirect regulation as no CsiR binding was seen.

#### NtrC and Nac

NtrC and Nac are the two principal regulators of nitrogen metabolism [64–66]. Nitrogen availability is sensed by monitoring levels of intracellular glutamine, which are linked to glutamate levels through nitrogen assimilation pathways (Additional file 1: Figure S5). Under low nitrogen conditions, the regulator NtrC is activated by phosphorylation by NtrB and modulates numerous σ-^54^ dependent genes. Consequently, Nac is induced and in turn modulates a set of σ-^70^ genes [54]. This coordinated activity of NtrC and Nac in low nitrogen affects components in the two major ammonia assimilation pathways: the glutamate synthase (GOGAT) pathway consisting of *glnA* and *gltBD*, and the glutamate dehydrogenase (GDH) pathway consisting of *gdhA*. NtrC activation leads to the glnA induction, while Nac represses gltBD [54]. Our data confirm all the previously known binding sites for Nac and identify numerous additional potential regulatory interactions (Additional file 1: Table S1, Additional file 3). As with CsiR, these include a surprising number associated with AR2 (Fig. 2). Our data also shows the reported repression of gltBD by Nac while also possibly repressing gdhA (Additional file 3, Fig. 2).

For Nac, we observe strong binding within the *gadE* gene, and both our RNA-seq data and published expression data for the perturbation of NtrC and Nac [54] indicate that Nac induction activates *gadE* expression (Table 2). Further confirmation of this binding site using ChIP-qPCR on natively tagged Nac in Gutnick media (see Methods) showed a 4.4-fold enrichment increase of the occupancy at the site within *gadE*. We also identify binding and apparent regulation by Nac for several genes in the circuitry upstream of GadE (Fig. 2). These data are consistent with previous reports indicating *gadBC* and *gadA* induction by acid in the absence of an σ-^s^ and potentially dependent on σ-^70^ [67, 68]. We identify two Nac binding sites associated with the *csiD*-*ygaF*-*gabD*-*gabT*-*gabP* operon. In addition to the previously reported regulatory site upstream of *gabD* [54], we also identify a site upstream of csiD. We further identify two binding sites associated with the *sucABCD*-*sdhCDAB* operon whose genes catalyze the TCA reactions between α-KG, succinate and fumarate (Additional file 2).

Our data also recapitulate the known regulatory interactions of NtrC, as noted above (Additional file 1: Table S1 and Figure S2–S4). Although different methods and conditions were utilized, a comparison of our data with ChIP-Seq of NtrC by Brown et al. [30] reveals substantial agreement between the two datasets and with previously biochemically identified binding sites (Additional file 1: Figure S2–S4). Our data refine binding sites reported in the previous manuscript and extend these results with additional detected sites (Additional file 4). In particular, we identify weak binding and apparent repression by NtrC of the *speF*/*potE* operon, the first potential direct regulatory link for ODAR identified. No direct binding of NtrC to elements of AR2 was detected.

#### OmpR

OmpR is a response regulator known to regulate several genes involved in osmotic stress adaptation [69, 70]. Recently, it was also shown that an OmpR mutant is unable to survive even mild acid stress [71]. OmpR is thought to be regulated by IHF, Crp, and ppGpp. We identify a novel Nac binding site in the divergent promoter between *ompR* and *greB*. Our gene expression analysis suggests this site may repress both genes (Additional file 5). Our ChIP-Seq mapping of OmpR detected all sites with experimental evidence for binding *in vivo*, verifies several sites for which there was no previous evidence of binding, and identifies 46 previously undetected sites (Additional file 1: Table S1, Additional file 5). EcoCyc also includes binding of OmpR to the promoter of *bolA* based on *in vitro* binding data [72]. However, this binding site was not detected in a more recent *in vivo* study [73], and we do also do not identify this site in our *in vivo* data.

Of note, the OmpR binding sites we identified include binding upstream of the *csiD*-*ygaF*-*gabD*-*gabT*-*gabP* GABA metabolism operon, and upstream of *yagU*, a gene coding for an inner membrane protein required for AR [24]. RT-PCR following induction of *ompR* resulted in a 2-fold increased expression of *yagU* compared to WT. A recent publication describing the ChIP-chip mapping of OmpR in both *E. coli* and *Salmonella typhimurium* reported binding of OmpR upstream of CadBA [73]. Our data do not support this conclusion (Additional file 1: Table S2).

### A Regulatory Network Linking Acid Resistance to Broader Cellular Metabolism

Collectively, our data suggest interactions between the regulation of different AR systems, GABA and glutamate metabolism, nitrogen metabolism, and the TCA cycle (Fig. 2, Additional file 1: Figure S5). This regulatory cross talk is mirrored in the known metabolic connectivity between these pathways (Fig. 2). These data suggest that Nac and CsiR may modulate AR, and their links to *gadE* and the network upstream of *gadE* suggest a role in AR2 specifically.

**Fig. 3:**
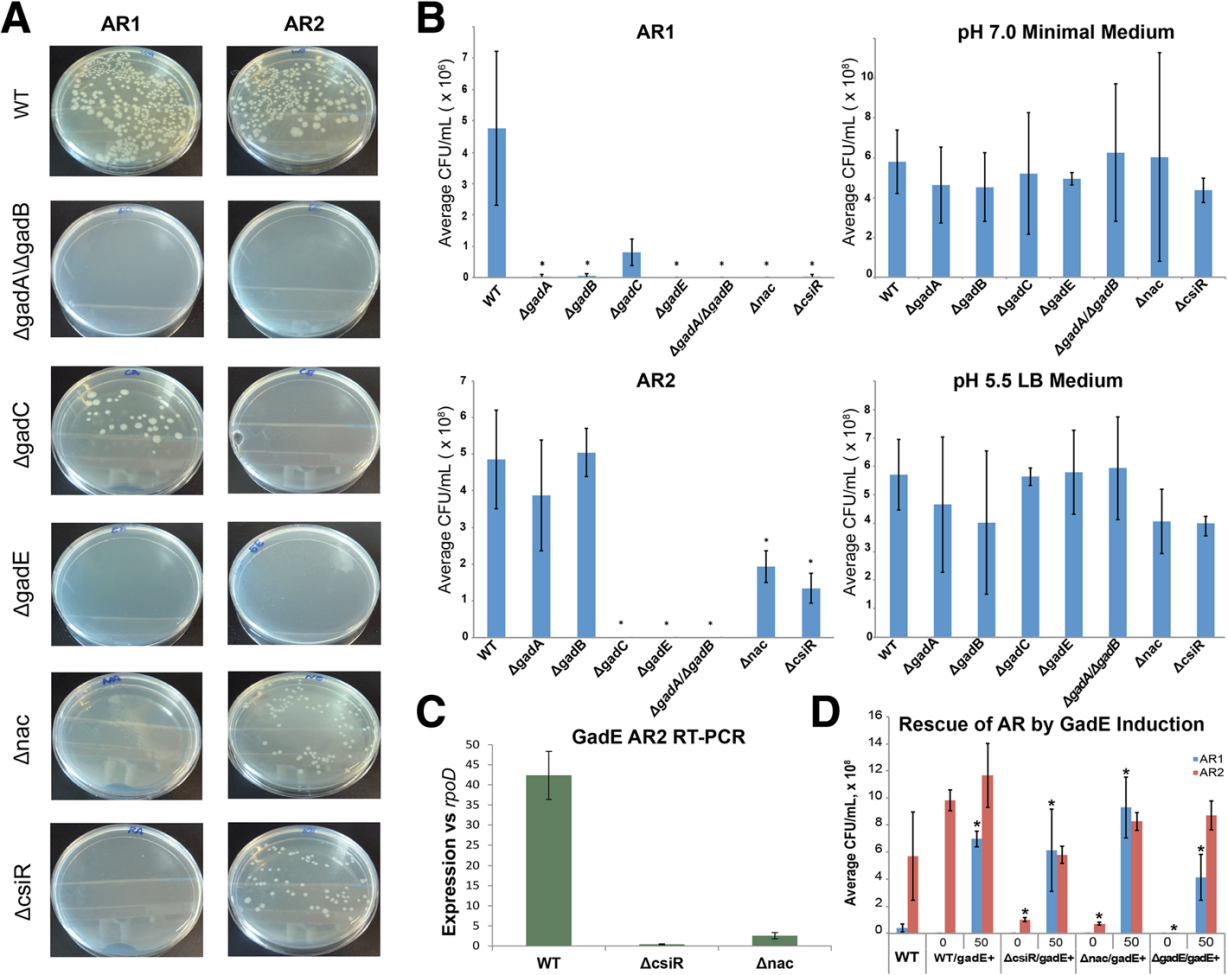
Validation of a Proposed Mechanism for AR1. We hypothesized that AR1 may be mediated by the AR2 machinery using an internal source of glutamate. Our regulatory network implicates both *nac* and *csiR* in this process. We tested this hypothesis by examining the phenotype of several deletion mutants in acid stress assays using published protocols for inducing AR1 or AR2, along with positive and negative controls (Castanie-Cornet et al. [11]; Lin et al [4]). Acid stress assays consisted of overnight culture, acid challenge at pH 2.5 for 2 h, followed by plating, overnight incubation, and colony counting (Methods). **a** Example plates for one experiment for selected mutants comparing AR1 conditions to AR2 conditions. **b** Summary of colony counts averages for all mutants across all experiments for AR1, AR2, and for two non-acidic control growth conditions (for which strains were plated directly after overnight incubation without acid challenge) for 3 replicates (*n* = 3). Colony counts provided to allow comparison to control WT data. Resulting counts were tested at a significance level of α = 0.05 (* *p*-value < 0.05). Plots of % survival for AR1 and AR2 are provide in Additional file 1: Figure S6 **c** RT-PCR of gadE in WT, *ΔcsiR*, and *Δnac* from colonies recovered after acid challenge following AR2 induction (*n* = 3 for all). **d** AR Rescue of KO strains via induction of gadE showing the summary of colony counts averages for WT, *ΔcsiR*, *Δnac* and *ΔgadE* with gadE induced in AR1 and AR2 conditions for 3 replicate experiments (*n* =3). Numbers on the x-axis above strain names indicate amount of aTc added during AR challenge in ng/μL. Resulting counts were tested at a significance level of α = 0.05 (* *p*-value < 0.05)

To test this possibility, we examined the phenotype of Δ*csiR* and Δ*nac* mutants in acid challenge under different AR conditions using well-described experimental protocols for inducing each system, along with corresponding positive and negative controls (Fig. 3a) [2, 7, 11, 14]. Single gene knockout strains were acquired from the Keio collection and sequence-verified [45]. Neither Δ*csiR* nor Δ*nac* displayed altered growth in standard non-acid conditions (Fig. 3b). However, when acid challenged in pH 2.5 after induction of AR2, both Δ*csiR* and Δ*nac* displayed significantly decreased colony recovery (Fig. 3b) and survival (Additional file 1: Figure S6). We further tested both strains in AR1-inducing conditions. Surprisingly, deletion of either *csiR* or *nac* fully abolished growth and survival under AR1 (Fig. 3b, Additional file 1: Figure S6).

### A Proposed Mechanism for AR1

The connectivity of the regulatory network, the experimentally confirmed impact of Δ*csiR* and Δ*nac* on both AR2 and AR1, and the metabolic connections between AR and central metabolism imply the possibility that AR under different conditions is modulated by both the intracellular and extracellular availability of key intermediates. This led us to a specific hypothesis concerning the mechanism for AR1. AR1 differs from other systems in that it does not require a specific external amino acid supplement. Given the connections between Nac, CsiR, AR2, and the internal metabolism of glutamate suggested by our data, we hypothesized that AR1 utilizes the decarboxylation mechanism of AR2 with an internal glutamate source. Our proposed mechanism for AR1 makes several specific and testable predictions. In particular, if AR1 uses the decarboxylation mechanism of AR2, it should require the decarboxylases GadA and/or GadB and the protein that induces these, GadE, but not require the glutamate transporter GadC.

To test these specific predictions we acquired and sequence-verified Δ*gadE*, Δ*gadA*, Δ*gadB*, and Δ*gadC* gene deletion strains from the Keio collection. We further generated a Δ*gadA*Δ*gadB* strain in which both AR2 decarboxylase genes were deleted since deletion of either *gadA* or *gadB* does not fully eliminate AR2 [11]. None of the deletions impacted growth in standard non-acid conditions (Fig. 3b). Moreover, all three AR2-associated genes are required for AR2, as expected (Fig. 3b). We see essentially no colony formation after extreme acid stress under AR2, although mutants have no impact in growth in neutral pH. AR2 also requires glutamate as expected. AR2 resistance is still present in Δ*gadA* or Δ*gadB*.

Consistent with our model for AR1, GadE and GadA/GadB are required for resistance in AR1-inducing conditions despite the absence of external glutamate during the overnight pre-incubation and the 2 h of acid stress (Fig. 3b). In addition, as predicted, GadC is not required for AR1 [11]. Although deletion of GadC decreased survival during AR1 relative to WT, all Δ*gadC* experiments for AR1 resulted in colonies while none for AR2 did. Moreover, deletion of either *gadA* or *gadB* individually was sufficient to render AR1 ineffective. None of the studied mutants impacted the neutral positive control, nor the AR1 specific negative control in which cells were pre-incubated with glucose. These data indicate that AR1 requires both the glutamate decarboxylation genes of AR2, and the primary regulator of these genes, but not the AR2 transporter for extracellular glutamate.

### GadE Expression Can Explain Δ*nac* and Δ*csiR* Phenotypes and Limited AR1 Efficacy

Given the model that AR1 utilizes an internal source of glutamate with the AR2 decarboxylase machinery, we hypothesized that both CsiR and Nac could modulate AR1 as a consequence of their regulation of AR2 via *gadE*. As described above, both *nac* and *csiR* induction increases *gadE* expression, with the former effect acting through direct binding of Nac to *gadE* while the latter presumably through binding and regulation of the circuit upstream of *gadE*. This implies that Δ*csiR* and/or Δ*nac* may impact AR by decreasing expression of GadE. We confirmed this experimentally in two ways.

First, we used RT-qPCR to measure *gadE* RNA in each of the deletion mutants relative to *rpoD* in colonies recovered after AR2 induction and acid stress. As shown in Fig. 3c, *gadE* expression was significantly decreased in both Δ*csiR* and Δ*nac*, as predicted, though not entirely abolished. As AR2 was also reduced but not abolished in these strains, the residual level of *gadE* expression appears sufficient to confer a degree of acid resistance via AR2. In contrast, the residual level of gadE expression in Δ*csiR* and Δ*nac* appears insufficient to support AR1.

Second, to confirm that decreased gadE expression levels are sufficient to explain the AR phenotypes of Δ*csiR* and Δ*nac*, we rescued AR in these strains by inducing *gadE* ectopically. We cloned *gadE* into an inducible vector under the control of the tetO operator. This vector was then introduced into ΔcsiR, Δ*nac*, Δ*gadE* and WT strains (see Methods). As shown in Fig. 3d, induction of *gadE* in ΔcsiR and Δ*nac* during acid challenge was capable of restoring WT levels of AR2 survival and providing substantial AR1 survival. Induction of *gadE* also restored WT levels of AR2 survival in the ΔgadE background, indicating that the functionality of induced *gadE* was not detectably altered.

Surprisingly, induction of *gadE* in ΔcsiR, Δ*nac*, and Δ*gadE* during AR1 conditions resulted in significantly more colony recovery than observed in WT strains (see Fig. 3d, *blue bars*). This suggests increasing GadE expression could increase the efficacy of AR1. This is supported by significantly increased colony recovery during AR1 when *gadE* was induced in a WT background. In contrast, AR2 survival was not substantially increased. Together these data confirm the role of *gadE* in both AR2 and AR1, indicate that decreased *gadE* expression is sufficient to explain the impact of ΔcsiR and Δ*nac* on AR2 and AR1, and suggest that *gadE* expression may be a limiting factor in AR1 but not AR2.

## Discussion

The primary transcriptional regulatory elements of amino acid-dependent AR have been characterized, but little is known about whether or how different system AR systems and adaptations are coordinated, or how AR is coordinated with broader cellular metabolism. We have utilized a combination of ChIP-Seq and transcriptomics to map the potential regulatory interactions of four transcriptions factors that appear to coordinate acid resistance, glutamate metabolism, and nitrogen metabolism: CsiR, Nac, NtrC, and OmpR. Taken together, our data suggest that the regulatory network underlying AR is complex and interconnected with the regulation central metabolism (Fig. 2, Additional file 1: Figure S5).

Our findings led to an experimentally confirmed mechanism for AR1. AR1 differs from other systems in that it does not require a specific external amino acid supplement. The network model inferred from our data implies that AR under different conditions is modulated by both the intracellular and extracellular availability of key intermediates. Given the connections between *nac*, *csiR*, AR2, and the internal metabolism of glutamate suggested by our data, we hypothesized that AR1 utilizes the decarboxylation mechanism of AR2 with an internal glutamate source. Although it has been proposed that internal GABA and glutamate metabolism might cooperate with the GadA and GadB decarboxylases in AR [55], this has not been confirmed, nor has it been linked to a mechanism for AR1. Our proposed mechanism for AR1 made several specific and testable predictions. In particular, if AR1 uses the decarboxylation mechanism of AR2, it should require the decarboxylases GadA and/or GadB and the protein that induces these, GadE, but not require the glutamate transporter GadC. We confirmed these predictions experimentally (Fig. 3).

Our experimental results also confirm the functional importance of the regulatory links we identified between *nac*, *csiR* and AR. Deletion of either *nac* or *csiR* substantially diminished the efficacy of AR2 during acid challenge, and abolished AR1. Our regulatory network model predicted that deletion of nac and csiR would decrease the expression of *gadE* during acid challenge, and we confirmed this via RT-PCR (Fig. 3c). We further confirmed that this decrease in gadE expression was sufficient to explain the AR phenotypes observed. Induction of gadE in ΔcsiR and Δ*nac* resulted in robust survival in both AR2 and AR1 conditions (Fig. 3d).

Induction of *gadE* in ΔcsiR and Δ*nac* restored AR2 survival to WT levels (Fig. 3c). In AR1 conditions by contrast, *gadE* induction in these backgrounds resulted in significantly more survival relative to WT. We also observed substantially greater AR1 survival when *gadE* was induced in a WT strain, while AR2 survival was not substantially increased. Thus, increasing *gadE* expression is sufficient to increase AR1 efficacy, but not AR2. This suggests that *gadE* expression may be limiting in AR1, but is not limiting in AR2. However, differences in gadE expression are not sufficient to fully explain the difference between AR2 and AR1 efficacy. At corresponding levels of gadE expression, we see consistently greater survival in AR2 relative to AR1. This was observed in both ΔcsiR and Δ*nac*, where the residual level of *gadE* expression was sufficient to confer measurable protection from AR2, but none from AR1. Similarly, *gadE* induction in all background strains tested resulted in greater survival in AR2 relative to AR1. We hypothesize that differences in the levels of intracellular glutamate available to *gadA*/*B* may explain part of these results.

Our data provide new perspective on previously published results. Although previous studies have demonstrated that deletion of either σ^54^ or *ntrC* leads to increased expression of GDAR and increased acid resistance [74, 75], the lack of direct interactions between NtrC and known regulators of AR2 suggests this effect is indirect. The role of Nac in AR2 suggests that part of this effect may be through the known σ^70^–dependent regulation of *nac* by NtrC. However, the activation of GDAR by *ΔntrC* cannot be easily explained by this link alone. Previous studies have also demonstrated that NtrC, RcsB, and GadX regulate the locus of enterocyte effacement (LEE) pathogenicity island in enterohemorrhagic *E. coli*, indicating that the coordination of nitrogen metabolism and AR can play both non-specific (through acid resistance) and specific (through LEE) roles in *E. coli* pathogenesis [74–77].

The link between AR1 and AR2, and the potential role for Nac and CsiR in mediating this link, raise many questions that remain to be investigated. First, questions remain about the mechanism of regulation of *gadE* by Nac. Our ChIP-Seq data reproducibly identifies binding of Nac to the GadE coding region (Fig. 1). Extensive genic binding has been previously reported for bacteria [28] and many experimentally confirmed examples of transcriptionally functional binding of TFs within coding regions in bacteria have been reported [59, 78–82]. Our RNA-Seq data (Tables 1 and 2) and previously published data [54] confirm that increased expression of Nac increases *gadE* mRNA levels. However, whether this change in mRNA levels is mediated through transcription initiation, transcription elongation, or mRNA stability remains to be determined. Second, if intracellular glutamate is the basis for AR1, as our hypothesis and data suggest, the source(s) of this glutamate remain to be determined. One speculative source is the conversion of α-KG from the TCA cycle to glutamate via GabD or GabT, possibly consistent with the regulation of the *suc*/*sad* operon by Nac [55]. Required maintenance of TCA cycle intermediates during growth on glucose may then contribute to the glucose repression of AR1. Third, what is the fate of GABA during AR1 if glutamate is being converted into GABA by GadA or GadB? We speculate that the decreased effectiveness of AR1 in the Δ*gadC* strain suggests the need to export GABA. Finally, the specific timing and roles of the newly identified regulatory links during acid, the roles of σ-^70^ and σ-^s^, and the role of non-transcriptional regulatory mechanisms also remain to be determined.

## Conclusions

We have presented a comprehensive genome-wide mapping of four TFs in *E. coli* using a combination of ChIP-Seq and transcriptomics: CsiR, Nac, NtrC, and OmpR. Our data identified all previously in vivo confirmed direct interactions and revealed several others previously inferred only from gene expression data. Our data also reveal novel regulatory interactions that appear to coordinate carbon and nitrogen metabolism with acid resistance. We have experimentally verified that CsiR and Nac, which are known carbon and nitrogen metabolism regulators respectively, modulate acid resistance through the transcriptional regulation of *gadE*, the master regulator of AR2. Our data also led to a model for the mechanism of the first described acid resistance mechanism, AR1. Our model predicts that AR1 utilizes the decarboxylation enzymes of AR2 but with internally derived glutamate. We have experimentally verified predictions arising from this model. Together our data provide new insight into the mechanisms of acid resistance in *E. coli*, and reveal an interconnected regulatory network that coordinates acid resistance with broader cellular metabolism.

## Additional files

Additional file 1: Supplementary text and materials of additional information presented in the paper. (DOCX 1478 kb)
Additional file 2: Peaks called and bound and gene expression data for CsiR. (XLSX 410 kb)
Additional file 3: Peaks called and bound and gene expression data for Nac. (XLSX 466 kb)
Additional file 4: Peaks called for NtrC. (XLSX 11.1 kb)
Additional file 5: Peaks called and gene expression data for ompR. (XLSX 28.0 kb)

## Abbreviations

ADAR: Arginine-dependent system
AR: Acid resistance
aTc: Anhydrotetracycline
ChIP: Chromatin-immunoprecipitation
EG: E-minimal with glucose
GABA: Gamma-aminobutyric acid
GDAR: Glutamate-dependent acid resistance system
GDH: Glutamate dehydrogenase pathway
GOGAT: Glutamate synthase pathway
IPTG: Isopropyl β-D-1-thiogalactopyranoside
LB: Luria-Bertani
LDAR: Lysine-dependent system
LEE: Locus of enterocyte effacement
MTB: *Mycobacterium* tuberculosis
ODAR: Ornithine-dependent acid resistance system
PCR: Polymerase chain reaction
RT-PCR: Reverse transcriptase PCR
TCA: The citric acid cycle
TF: Transcription factor
WT: Wild-type
