# Supplementary material for: Coordinated regulation of acid resistance in Escherichia coli
Source: BMC Syst Biol. 2017 Jan 6;11:1. doi: 10.1186/s12918-016-0376-y (PMC5217608; doi:10.1186/s12918-016-0376-y)
Supplement: Supplementary file 1 [file 12918_2016_376_MOESM1_ESM.docx]

# Additional file 1

# Validation of Binding Site Mapping

# Our regulatory network mapping strategy utilized transcription factors tagged with FLAG and under inducible control (Methods) [28, 42, 57-60]. The use of FLAG-tagged TFs obviates the need for antibodies to each native protein.  For immunoprecipitation (IP) we are able to use an extensively validated ChIP-grade antibody to FLAG that provides high sensitivity and specificity.  Control IP experiments in strains lacking FLAG-tagged proteins revealed minimal non-specific binding in E. coli.  The use of an inducible promoter system ensures expression of targeted TFs, which allowed us to study the binding of all TFs in the same standard reproducible condition.  Induction also results in a transient perturbation of each TF.  RNA-Seq following TF induction thus provides a global view of the regulatory functions of each TF that can be directly compared to the binding profile for the same TF in the same condition. While the induction of TFs raises concerns about overexpression artifacts, we have extensively validated this approach on over 100 TFs in Mycobacterium tuberculosis [42, 57-59], and related Mycobacteria. We also confirmed the accuracy of this approach in E. coli for the TFs studied in this report in several ways.

# First, we compared our results to previously experimentally confirmed binding sites in EcoCyc.  Binding sites in EcoCyc include sites for which there is direct experimental evidence of binding, and sites that are only inferred by gene expression data or computational approaches.  As documented in Table S1, we identified all binding sites for Nac and NtrC for which there is direct evidence of binding, and we identified all 6 binding sites for OmpR with direct evidence of binding in vivo. No binding sites for CsiR with direct experimental evidence have been reported. Our data also confirm a large number of binding sites that were previously only inferred by gene expression data (Table S1).  Second, we confirmed the spatial accuracy and reproducibility of our ChIP-Seq data as compared with previously identified sites.  As shown in Figure 1 and Table S1, our data show extremely high spatial accuracy, with the majority of predicted sites within 20 nucleotides of known sites and sufficient resolution to resolve multiple binding sites within individual enriched regions (Figure 1,Table S1).

# Third, we confirmed that we recovered the correct binding motif for TFs where this has been previously reported (Figure S1).  Potential binding motifs for Nac, NtrC, and OmpR have been reported, while CsiR lacks any previously identified binding sites on which to base a motif.  We identified binding motifs for all four TFs based on the complete set of binding regions (see Methods). For Nac, an ATA-N9-TAT consensus binding sequence has been first reported in K. aerogenes [83] and Nac in E. coli was subsequently shown to bind to ATA-N9-TAT and ATA-N9-GCT promoter sequences [84]. Consistent with these reports, we identify a clear ATA-N9-TAT motif within our data.  We also identify other more weakly informative bases in the motif (Figure S1).  For NtrC, a preliminary consensus binding sequence in S. typhimurium was reported as TGCACC – N4 – TGGTGCA [85]. Based on our E. coli data, we identify a motif with a clear TGCAC–N7–GTGCA matching the core palindromic bases on the S. typhimurium motif.  While our motif also suggests informative bases within the N7 region that include the bases identified in S. typhimurium, our data suggests that these bases are not as well conserved in the E. coli motif. For OmpR, several candidate binding sequences have been proposed.  An in vitro analysis of OmpR binding to synthetic oligonucleotides was used to propose a consensus sequence of TTTTACTTTTTGTAACATAT [86].  An in vivo DNAse footprinting analysis of the OmpR binding sites to ompF, and ompC was used to generate the consensus sequence TTAC [87], while a subsequent study from the same lab based on the analysis of OmpR binding to ompF revealed strongest binding to a sequence consisting of two direct repeats of TTACATNTN.  Consistent with these reports, our analysis of all OmpR binding sites predicted from ChIP-Seq generated a core binding motif that includes a two direct repeats of TTACA.

# Fourth, we compared our results with previously published ChIP-Chip data for OmpR [73] and ChIP-Seq data for NtrC that were published during the preparation of this manuscript [30].  Quinn 2014 [73] performed ChIP-Chip on FLAG tagged OmpR in both Salmonella Typhimurium serovar Typhimurium and E. coli.  We compared binding sites identified by Quinn in E. coli to our results and previously identified sites in E. coli in EcoCyc (Table S2). Where ChIP-Chip identified previously known binding sites in EcoCyc, our data also identifies these sites.  In addition, our ChIP-Seq data identify a larger number of previously verified binding sites from EcoCyc.

# Brown (2014) performed used ChIP-Seq with FLAG-tagged NtrC to identify global binding sites [30].  NtrC in this study was induced from the native promoter by nitrogen starvation, and ChIP-Seq results were compared to experiments performed with nitrogen in the medium.  We compared these results with our results and known binding sites for NtrC from EcoCyc to directly assess the impact of using an inducible promoter versus the native promoter for this factor.  As shown in Figure S2 our results identify all EcoCyc binding sites that were also identified by Brown (2014).  In addition, we identify one previously reported binding site not identified by Brown study.  Comparing all binding sites identified by Brown (2014) with our data (Figure S3) reveals nearly complete agreement on bound regions.  Three regions reported by Brown were not called in our results and a closer examination reveals no apparent shift in forward and reverse read coverage in data from the published study, suggesting that these reported sites are likely false positives.  The major difference between our data and the previously published data is in peak enrichment.    Peak enrichment relative to genomic background was an average of 16-fold greater in our study versus the Brown study (Figure S4).  This high enrichment led to the identification of additional weaker-affinity binding sites and contributed to the high accuracy of identifying known binding site locations from EcoCyc (Figure S3).  

# Supplementary Figures and Tables

Table S. Comparison of ChIP-Seq data with reported regulatory interactions in EcoCyc.  Each row represents a reported regulatory interaction from EcoCyc. In some cases, the regulatory interaction is based on evidence for direct binding, in other cases only on expression data, as noted in the last column.  Corresponding binding sites from ChIP-Seq data are shown where identified.   Coverage is the read coverage at the position of maximum enrichment normalized mean coverage over the entire genome.

| EcoCyc Binding Sites | Chip-Seq Peaks | Notes |
| --- | --- | --- |
| TF | Target Operon | Location(s) | Peak Location | Dist to Start | Dist to Known | Coverage |  |
| nac | asnC-mioC-mnmG | 3925061, 3925118, 3925039 | 3925040 | -14 | 1 | 315 | No previous evidence of direct binding |
| nac | codBA | 354050 | 354012 | -134 | -38 | 35 |  |
| nac | gabDTP | 2789150 | 2789145 | -150 | -5 | 20 | No previous evidence of direct binding |
| nac | gdhA | 1840307, 1840356, 1840376, 1840251 | 1840378 | -17 | 2 | 8 | No previous evidence of direct binding |
| nac | gltBDF | N/A | 3352237 | -510 |  | 42 | No previous evidence of direct binding |
| nac | nac | 2060077 | 2060104 | -147 | 27 | 75 |  |
| nac | nupC | N/A | 2510922 | -142 |  | 162 | No previous evidence of direct binding |
| nac | serA | 3056508, 3056497 | 3056505 | -73 | -3 | 144 | No previous evidence of direct binding |
| nac | ydcSTUV-patD | 1509623 | 1509484 | -194 | -139 | 163 |  |
| ompr | dtpA | 1710695 | 1710652 | -141 | -43 | 41 |  |
| ompr | ompC | 2310941, 2310900, 2310920 | 2310952 | -181 | 11 | 107 |  |
| ompr | flhDC | 1976402, 1976565 | 1976385 | -164 | -17 | 16 | No previous evidence of direct binding |
| ompr | ompF | 986686, 986406, 986366, 986386 | 986408 | -203 | 2 | 148 |  |
| ompr | fadL | 2459078, 2459296, 2459346, 2459167 | - | - |  | - | No direct evidence of binding |
| ompr | csgDEFG | 1102617 | 1102629 | -210 | 12 | 93 |  |
| ompr | nmpC | N/A | - | - |  | - | No direct evidence of binding |
| ompr | bdm-sra | N/A | - | - |  | - | No direct evidence of binding |
| ompr | ecnB | 4374532 | - | - |  | - | No direct evidence of binding |
| ompr | omrB | 2974479, 2974479 | 2974473 | -66 | -6 | 9 | No previous evidence of direct binding |
| ompr | omrA | 2974479, 2974479 | 2974473 | -66 | -6 | 9 | No previous evidence of direct binding |
| ompr | micF | 2310920, 2310941, 2310900 | 2310952 | -154 | 11 | 107 |  |
| ompr | bolA | 453611 | - | - |  | - | Evidence of in vitro binding Yamamoto (2000). Not identified by in vivo assay from Quinn (2014) |
| ntrC | ddpXABCDF | 1561219, 1561240 | 1561224 | -124 | 5 | 23 |  |
| ntrC | astCADBE | 1830301, 1830343, 1830321 | 1830313 | -307 | -8 | 49 |  |
| ntrC | yeaGH | 1864932, 1866979 | - | - |  | - | No previous evidence of direct binding |
| ntrC | patA | N/A | 3217292 | -224 |  | 56 | No previous evidence of direct binding |
| ntrC | yhdWXYZ | 3416898, 3416868 | 3416958 | -213 | 60 | 66 |  |
| ntrC | argT-hisJQMP | 2425954, 2426061 | 2426105 | -292 | 44 | 127 |  |
| ntrC | glnA | 4056175, 4056198, 4056270, 4056238, 4056219 | 4056178 | -121 | 3 | 2520 |  |
| ntrC | astCADBE | 1830301, 1830343, 1830321 | 1830313 | -307 | 12 | 49 |  |
| ntrC | rutABCDEFG/rutR | 1073394, 1073373 | 1073395 | -161 | 1 | 18 |  |
| ntrC | potFGHI | N/A | - | - |  | - | No previous evidence of direct binding |
| ntrC | glnHPQ | 847405, 847392, 847349, 847379 | 847400 | -173 | -5 | 980 |  |
| ntrC | nac | 2060154 | 2060143 | -186 | -11 | 38 |  |
| ntrC | glnK-amtB | 471693 | 471706 | -116 | 13 | 107 |  |
| ntrC | cbl | 2057988 | - | - |  | - | No previous evidence of direct binding |

Table S. Comparison of OmpR binding sites from ChIP-Seq data (this study), ChIP-Chip data (Quinn 2014), and EcoCyc. The “X”s indicate evidence for binding from that source.  ChIP-Seq identifies a larger number of previously verified binding sites.  Many reported binding sites from ChIP-Chip appear to be false positives, although in two cases ChIP-Seq identifies binding further upstream of regions reported by ChIP-Chip, likely reflecting the greater noise and lower spatial resolution of ChIP-Chip relative to ChIP-Seq.  

| Locus | EcoCyc | ChIP-Chip | ChIP-Seq | Notes |
| --- | --- | --- | --- | --- |
| dtpA | X | X | X |  |
| ompC | X | X | X |  |
| flhDC | X | X | X |  |
| ompF | X | X | X |  |
| fadL | X |  |  | No direct evidence for binding |
| csgDEFG | X | X | X |  |
| nmpC | X |  |  | No direct evidence for binding |
| bdm-sra | X |  |  | No direct evidence for binding |
| ecnB | X |  |  | No direct evidence for binding |
| omrB | X |  | X |  |
| omrA | X |  | X |  |
| micF | X |  | X |  |
| bolA | X |  |  |  |
| elfA |  | X |  |  |
| rhsA |  | X |  |  |
| rhtA |  | X | X |  |
| alaA |  | X |  |  |
| lsrK |  | X |  |  |
| rseX |  | X |  |  |
| yfgF |  | X |  |  |
| dgsA |  | X |  |  |
| yaf |  | X |  |  |
| gltA |  | X |  |  |
| yehA |  | X |  | ChIP-Seq identifies binding in genes upstream |
| sstT |  | X | X |  |
| exuR |  | X |  |  |
| yiaG |  | X |  |  |
| yibB |  | X |  |  |
| aspA |  | X |  |  |
| CadB |  | X |  |  |
| yhjB |  | X | X | ChIP-Seq identifies binding in genes upstream |

 

| TF | Motif Sequence | Width | E-value |
| --- | --- | --- | --- |
| Nac |  | 17 | 4.4e-236 |
| ntrC |  | 18 | 2.0e-045 |
| csiR |  | 15 | 8.3e-095 |
| ompR |  | 20 | 5.1e-041 |

Figure S. Motifs identified for each Transcription Factor.  Binding motif sequences were generated using MEME SUITE tool (version 4.10.2) [[77](#_ENREF_77)] using a 4th-order markov model based on all identified binding regions for each TF.

Figure S. Comparison of NtrC ChIP-Seq data between this study and Brown et al. (2014) for binding sites reported in EcoCyc. As with Error: Reference source not found, each panel plots the total read coverage (blue), forward read coverage (green), and reverse read coverage (red). The maximum coverage for each plot is given by the number on the y-axis in units of coverage normalized to mean coverage. In each instance, data for this study (labeled ntrC) and data from Brown et al. (2014) for N-starved (ntrC N-) and N-non-starved (ntrC N+) conditions are shown are shown in the first, second, and third row of coverage from the bottom.  The location of binding sites for NtrC from EcoCyc displayed at black boxes in the ntrC track. The experiment of this study resulted in peaks with 16-fold greater coverage relative to background than the previously published results.  More symmetric peaks were also observed in this study.

Figure S. Comparison of NtrC ChIP-Seq data between this study and Brown et al. (2014) for binding sites reported in Table 1 of Brown et al. (2014). Data plotted as described in Figure S. In a small number of instances (3), the data suggest that previously reported sites may reflect false positives:  no apparent shift between forward and reverse read coverage is apparent in the previous data in these instances, and the sites were not reflected in our experiments.

Figure S. A comparison of enrichment for between our data and that of Brown et al. (2014) for sites identified in this study.  Coverage relative to background (median coverage) was 16-fold greater in this study, resulting in the identification of additional sites.

Figure S. Metabolic links between different AR systems, central metabolism, and nitrogen assimilation. Metabolic map reconstructed from selected pathways whose metabolites are linked with AR systems.  The map is not intended to be complete.  Metabolites in read are core metabolites for the amino-acid dependent AR systems.  Both primary nitrogen assimilation pathways are depicted within the dotted lines.

Figure S. Survival of deletion mutants after acid stress for AR1 and AR2. The CFU results of Error: Reference source not found plotted as percent survival for N=3 replicates.  Percent survival was determined as the percentage of colonies when platting after acid stress relative to the number of colonies from the same volume of overnight culture plated directly without acid stress. 

| (A) | (B) |
| --- | --- |

FIgure S. Measurement of TF expression levels during induction in strains used. (A) RT-PCR of TF expression levels in the different strains after artificial induction with 1 mM IPTG for two hours (n=3; see Methods). Conditions for physiological induction are as follows: For Nac and NtrC physiological induction, E. coli MG1655 WT strains were grown on N-C- minimal media supplemented with 0.4% glucose to mid-exponential phase (OD600 = 0.5). 5 mM glutamine was added as control [[51](#_ENREF_51)]; For CsiR physiological induction, E. coli MG1655 WT strains were grown on LB media to lag and mid-exponential phases (OD600 0.1 and 0.5 respectively). The sample in lag phase served as control [[52](#_ENREF_52)]; For OmpR physiological induction, E. coli MG1655 WT strains were grown on LB media mid-exponential phase (OD600 = 0.5) with 20% sucrose. A sample without sucrose was used as control [[82](#_ENREF_82)]. (B) Ratio of TF expression comparing artificially induced levels to physiologically induced TF levels.
